# Impact of gender and dialysis adequacy on anaemia in peritoneal dialysis

**DOI:** 10.1007/s11255-016-1499-1

**Published:** 2017-01-05

**Authors:** Alicja Ryta, Michal Chmielewski, Alicja Debska-Slizien, Piotr Jagodzinski, Malgorzata Sikorska-Wisniewska, Monika Lichodziejewska-Niemierko

**Affiliations:** 1 Department of Nephrology, Transplantology and Internal Medicine, Medical University of Gdańsk, Gdańsk, Poland; 2 Department of Hygiene and Epidemiology, Medical University of Gdańsk, Gdańsk, Poland; 3 Department of Palliative Medicine, Medical University of Gdańsk, Gdańsk, Poland

**Keywords:** Peritoneal dialysis, Gender, Dialysis adequacy, Anaemia, Erythropoiesis-stimulating agents

## Abstract

**Purpose:**

In the general population, haemoglobin (Hb) concentration is higher in men than in women. However, target Hb levels in dialysis patients are set constant regardless of the patient’s sex. The aim of this study was to evaluate Hb concentration and the use of erythropoiesis-stimulating agents (ESA) in peritoneal dialysis (PD) patients taking gender and dialysis adequacy into account.

**Methods:**

The study comprised two parts. The first was a cross-sectional analysis of Hb and ESA in 2180 prevalent PD patients. The second included 88 incident PD patients, followed for 36 months. During this time, the major parameters recorded at 12-month intervals included: Hb concentration, weekly ESA, total, renal, and peritoneal Kt/V. Erythropoietin resistance index (ERI) was calculated as the ratio between ESA dose and achieved Hb.

**Results:**

In prevalent PD patients, Hb concentration was significantly lower in women, (11.2 ± 1.4 vs. 11.5 ± 1.6 g/dl; *p* < 0.001), despite higher doses of ESA (2691 ± 1821 vs. 2344 ± 1422; *p* = 0.001). Hb concentrations were related to dialysis adequacy in both cohorts. However, despite significantly higher Kt/V, women were characterized by a lower Hb level. In incident patients, this association was present throughout the observation period, while the ESA dose in women was significantly higher at every time point. In multiple regression analysis, gender was an independent determinant of ERI (*b* = 0.34; *p* < 0.05).

**Conclusions:**

Despite higher dialysis adequacy, Hb concentration in women treated with PD is significantly lower, and the ability to correct it impaired, as compared to men.

## Introduction

 Anaemia is a constant complication of end-stage renal disease (ESRD). Driven mainly by a relative deficit in erythropoietin, it has an important impact on patient’s physical condition and quality of life. Vast majority of patients receive erythropoiesis-stimulating agents (ESA) to partially correct it. The target haemoglobin (Hb) concentrations are set constant, regardless of the patient’s sex [1]. This stays in contrast to the situation in the general population where Hb level is typically higher in men than in women. By current criteria, women are considered to be anaemic if their haemoglobin is less than 11.5 or 12.0 g/dl, while in males the threshold is set at 13.0 or 13.5 g/dl [2]. These gender-associated disparities in Hb concentration are believed to be due to several factors including sex hormones, iron deficiency due to menstrual losses, and perhaps poor nutritional intake [2]. These factors are, to some extent, absent in peritoneal dialysis (PD) patients. However, it is not clear whether this would result in equal Hb levels and similar ESA requirements in men and women on PD.

One of the major determinants of anaemia severity and ESA responsiveness is dialysis adequacy [3]. Patients with an inadequate dialysis dose are resistant to ESA, and their target Hb is harder to reach [4]. In haemodialysis, there is an inverse relationship between the achieved Kt/V and ESA dose, while increasing the dialysis dose is associated with an increase in haematocrit level [5, 6].

The primary aim of the present study was to evaluate potential associations between gender and Hb concentrations and ESA demand in prevalent as well as in incident PD patients. The second objective was to evaluate the potential associations between dialysis adequacy, assessed by the Kt/V, and Hb levels, as well as responsiveness to ESA treatment, taking gender into account.

## Materials and methods

The first part of the study was performed on the basis of the national PD Registry, on 2180 prevalent PD patients. This was a cross-sectional evaluation of Hb concentration and ESA dose in men and women treated with PD. Obtained data also included the prevalence of co-morbidities and the total weekly Kt/V. The Polish PD Registry was established by the National Consultant in Nephrology, and it replaced PD survey in which aggregated data were gathered from all PD centres in Poland. The basic individual patient and treatment data are collected by the Registry from all the PD centres in Poland on an annual basis. The diagnosis of co-morbidities was based on the information provided by the centres.

The second part was undertaken to get deeper insight into the mechanisms relating gender and dialysis adequacy to anaemia and ESA requirements of PD subjects. It was designed as a retrospective observational evaluation. All patients who initiated PD as their first renal replacement therapy (RRT) in a nephrology department of a large university-based hospital in the period between 2009 and 2013 were included. The typical dialysis prescription at baseline was four exchanges per day with a 2000 ml low-glucose solution for patients treated with continuous ambulatory PD (CAPD), and 10,000 ml of low-glucose fluid overnight for subjects who started on automated PD (APD; Fresenius, Bad Homburg, Germany). All patients were followed until death, kidney transplantation, transfer to haemodialysis, or the end of the follow-up period, with a maximum follow-up of 36 months since the start of dialysis. During this time, the evaluated parameters were recorded at 12-month intervals. These included: Hb concentration, weekly ESA dosage per kg of body mass, total weekly Kt/V, renal Kt/V, and peritoneal Kt/V. Most patients received erythropoietin-beta as their ESA (NeoRecormon; Roche Pharma AG; Grenzach-Wyhlen, Germany). For comparisons, the weekly dose of ESA in patients taking other ESA than erythropoietin-beta was converted into erythropoietin-beta units. The erythropoietin resistance index (ERI) was calculated as the ratio between the weekly weight-adjusted ESA dose and Hb concentration.

All biochemical analyses were performed using routine methods at the local laboratory. Apart from the abovementioned, they included: urea, creatinine, albumin, C-reactive protein (CRP), iron, ferritin, transferrin saturation. Diagnosis of cardiovascular disease (CVD), diabetes mellitus (DM), and arterial hypertension was made based on patients’ medical records. Glomerular filtration rate (GFR) was estimated based on the MDRD equation.

Protocol of the study received approval from the Local Bioethics Committee. Results are expressed as percentages (for categorical variables), mean and standard deviation, or median and interquartile range, as appropriate. The assumption of normality was verified with the Kolmogorov–Smirnov test. A *p* value <0.05 was considered to be statistically significant. Comparisons between two groups were assessed with a Student’s *t* test or a Mann–Whitney test, as appropriate. To assess correlations among the evaluated variables, Pearson’s correlation coefficient (*r*) was used. Independent associations among variables were assessed with stepwise multiple regression analysis. Statistical processing of the results was performed with the use of the statistical software STATISTICA PL v 12.0 (Statsoft, Kraków, Poland).

## Results

The cross-sectional evaluation of the data from the national PD Registry was based on 2180 prevalent PD patients. The group included 1023 women and 1157 men. Their clinical characteristics are presented in Table 1. Men were characterized by a higher prevalence of co-morbidities and a poorer dialysis adequacy, as compared to women. In the studied population, hypertensive patients had a lower Hb concentration, when compared to subjects with normal blood pressure (11.2 ± 1.6 vs. 11.4 ± 1.8 g/dl; *p* < 0.05). Lower Hb in hypertensive subjects did not result from drugs affecting the renin–angiotensin–aldosterone system (RAAS), as Hb level was identical in patients with and without RAAS blockers (11.3 ± 1.6 vs. 11.3 ± 1.7 g/dl, respectively). There was a weak but significant positive association between the total Kt/V and Hb level (*r* = 0.13; *p* < 0.05), and a considerable negative association between the achieved Kt/V and the required ESA dose (*r* = −0.19; *p* < 0.05). When Hb concentration was evaluated taking gender into account, it turned out to be significantly higher in men than in women (11.5 ± 1.6 vs. 11.2 ± 1.4 g/dl; *p* < 0.001). In multiple regression analysis, gender was among the independent predictors of Hb level, together with Kt/V, presence of diabetes mellitus and hypertension (combined *R* ^2^ = 0.31, *p* < 0.001). Percentage of men treated with ESA was significantly lower than women (69 vs. 76%; *p* < 0.001), and similarly, the average weekly dose of ESA was lower, as compared to women (2344 ± 1422 vs. 2691 ± 1821; *p* = 0.001).

**Table 1:** Baseline characteristics of the 2180 prevalent PD patients

	Men (*n* = 1157)	Women (*n* = 1023)	*p* value
Age	61 (48–71)	61 (47–72)	NS
DM (%)	33	29	0.03
Hypertension (%)	83	75	0.001
GN (%)	20	15	0.001
ADPKD (%)	4	6	0.03
Kt/V	2.25 ± 0.67	2.47 ± 0.74	0.001
Albumin (g/l)	32.1 ± 13.8	31.4 ± 13.9	NS

*DM* diabetes mellitus, *GN* primary glomerulonephritis, *ADPKD* autosomal dominant polycystic kidney disease

The second part of the analysis included a total of 88 patients who started PD treatment as their initial RRT in 2009–2013. This group comprised 53 men and 35 women. The primary kidney disease was primary glomerulonephritis in 25 patients (28%), diabetes mellitus in 15 subjects (17%), hypertensive nephropathy in 5 (6%), ADPKD in 5 (6%), other and unknown in 38 patients (43%). The prevalence of particular primary kidney diseases was comparable between men and women. Dialysis modality included CAPD in 67 patients (76%) and APD in 21 subjects (24%) and did not differ between genders. The baseline clinical characteristics of the studied cohort are shown in Table 2. Men were slightly older and had a higher prevalence of co-morbidities, as compared to women. At baseline, they also had an increased CRP, phosphate, and their body mass index (BMI) was significantly higher than in women. Neither of these variables turned out as significant predictors of Hb concentration in the multivariate analysis. The iron status was similar in both groups. Out of 53 men included in the study, ten (19%) were on oral iron supplementation with a standard dose of 100 mg of iron sulphate per day. In women, five (14%) received iron at baseline (*p* = NS). The percentage of patients prescribed iron increased during the study period and reached 47% in men and 55% in women after 3 years of follow-up (*p* = NS).

**Table 2:** Baseline characteristics of the 88 incident PD patients

	Men (*n* = 53)	Women (*n* = 35)	*p* value
Age (years)	57 (44–67)	50 (35–64)	NS
CVD (%)	47	26	0.05
DM (%)	34	31	NS
Hypertension (%)	100	94	NS
Body mass (kg)	79.6 ± 11.1	62.1 ± 12.5	0.01
BMI	25.3 ± 3.3	23.3 ± 4.5	0.01
Albumin (g/l)	37.3 ± 3.9	37.06 ± 3.6	NS
hsCRP (mg/l)	3.85 (1.60–8.20)	1.40 (0.70–3.80)	0.01
Iron (mcg/dl)	72.9 ± 24.5	64.7 ± 25.2	NS
Ferritin (ng/ml)	166 (83–211)	140 (73–332)	NS
TSAT (%)	27.0 ± 11.2	26.2 ± 12.0	NS
PTH (pg/ml)	330 ± 241	349 ± 257	NS
Ca (mg/dl)	8.92 ± 0.68	8.85 ± 0.76	NS
P (mg/dl)	5.39 ± 1.48	4.71 ± 1.28	0.03
RRF (ml/day)	1423 ± 652	1205 ± 594	NS

*CVD* cardiovascular disease, *DM* diabetes mellitus, *BMI* body mass index, *hsCRP* high-sensitivity C-reactive protein, *TSAT* transferrin saturation, *PTH* parathyroid hormone, *Ca* calcium, *P* phosphate, *RRF* residual renal function

Men started dialysis at a higher eGFR, although this difference was not statistically significant (9.7 ± 3.8 vs. 8.6 ± 3.2 ml/min/1.73 m^2^).

**Fig. 1:**
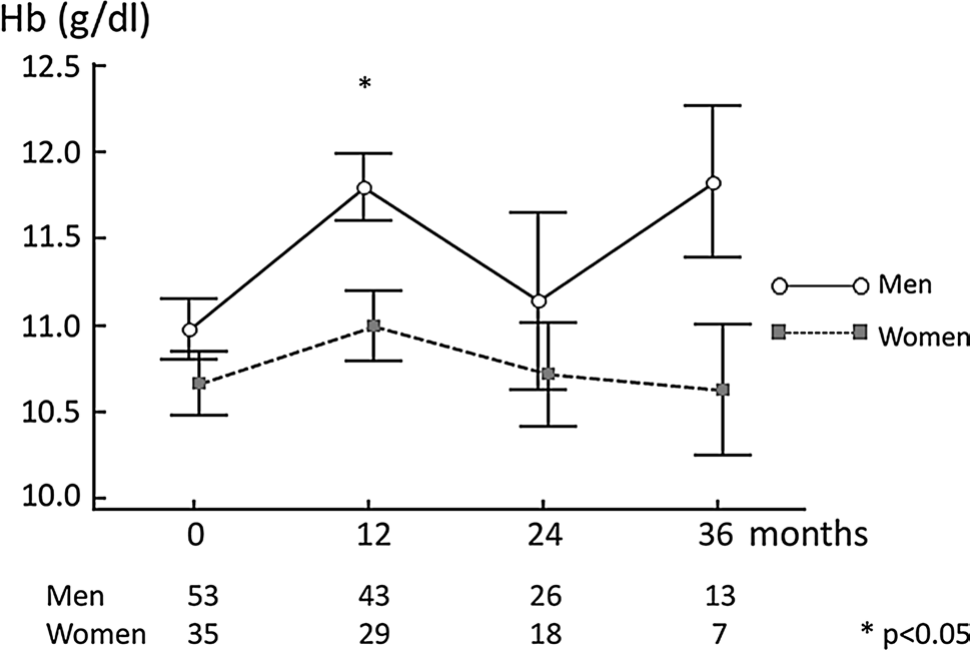
Haemoglobin concentration in 88 incident PD patients

**Fig. 2:**
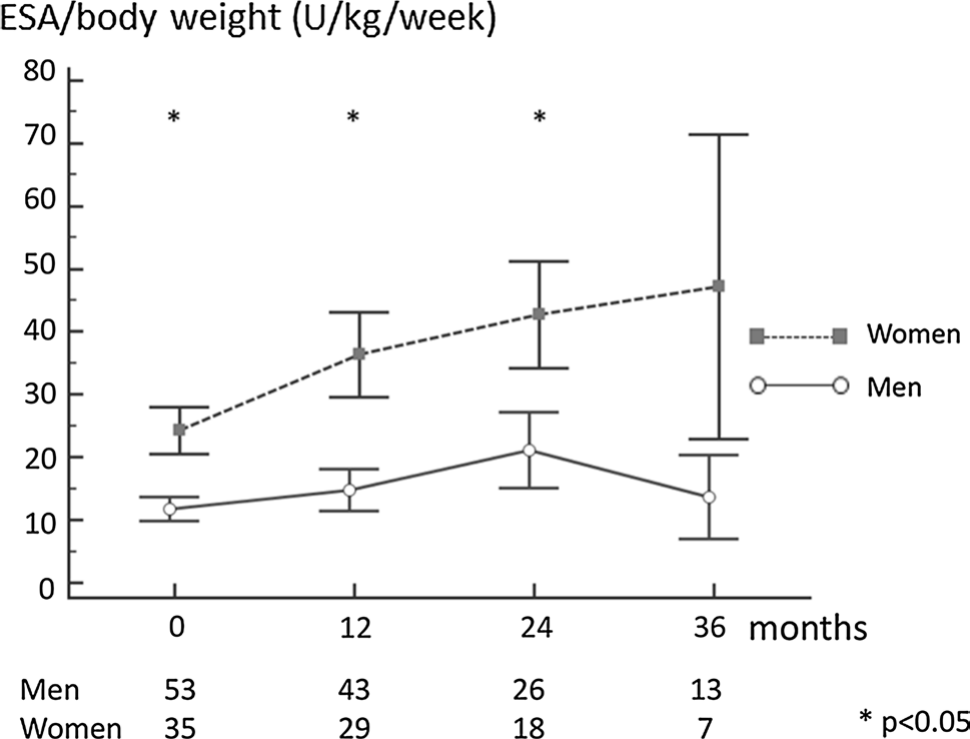
Dose of ESA; *ESA* erythropoiesis-stimulating agents in 88 incident PD patients, *BW* body weight

The baseline Hb concentration was lower in women, as compared to men, similarly to the situation observed in prevalent PD patients, and in the general population; Fig. 1. It has to be underlined that, at that time point, ten women (29%) and only three men (6%) received ESA as a pre-dialysis treatment. The difference in Hb concentration persisted throughout the 36-month observation period, varying with time, being most significant after 1 year of dialysis therapy (11.0 ± 1.1 vs. 11.8 ± 1.3 g/dl; *p* < 0.01). The lower Hb level in women was probably the major reason for high ESA doses prescribed. The mean doses remained fairly stable in men during the 3-year follow-up, while they constantly increased in women; Fig. 2. Similarly, ERI, calculated as the ratio between the ESA dose per body weight and achieved Hb, was higher in women, as compared to men. At baseline, the median ERI equalled 2.81 (1.82–3.78) in women versus 2.06 (1.37–2.94) in men (*p* = 0.04). In multiple regression analysis, following inclusion of: age, co-morbidities, CRP, albumin, iron use, and Kt/V, gender was an independent determinant of ERI (*b* = 0.34; *p* < 0.05). At the end of the 36-month observation period, the median ERI was 5.66 (2.85–6.16) in women versus 3.06 (1.46–4.88) in men, and this difference did not reach statistical significance due, mainly, to the low number of participants at that time point.

**Fig. 3:**
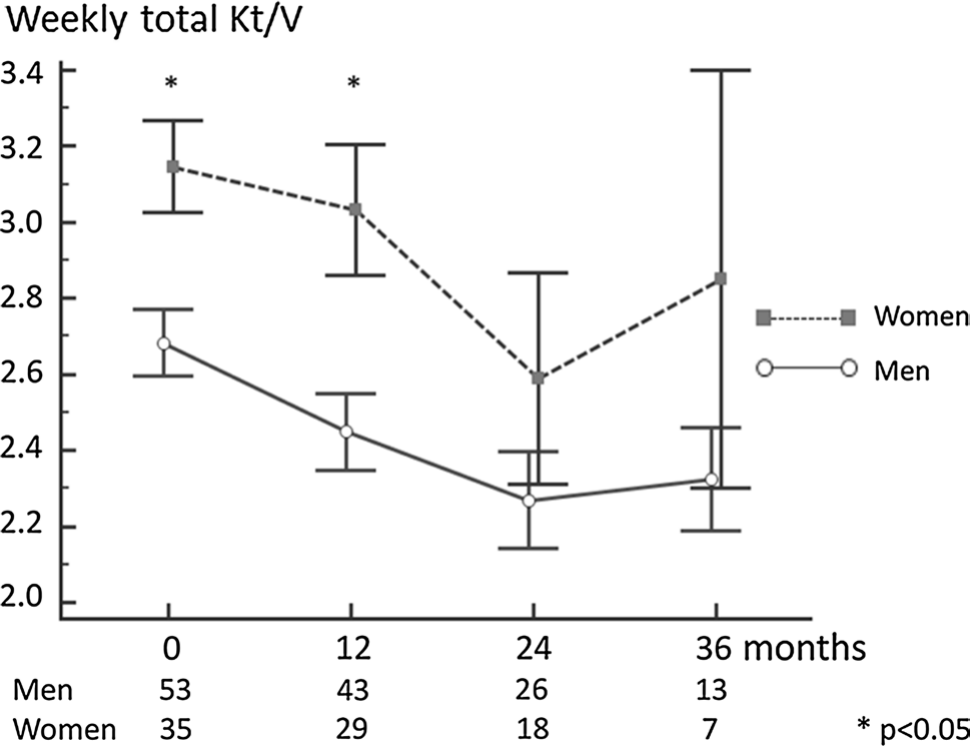
Weekly total Kt/V in 88 incident PD patients

**Fig. 4:**
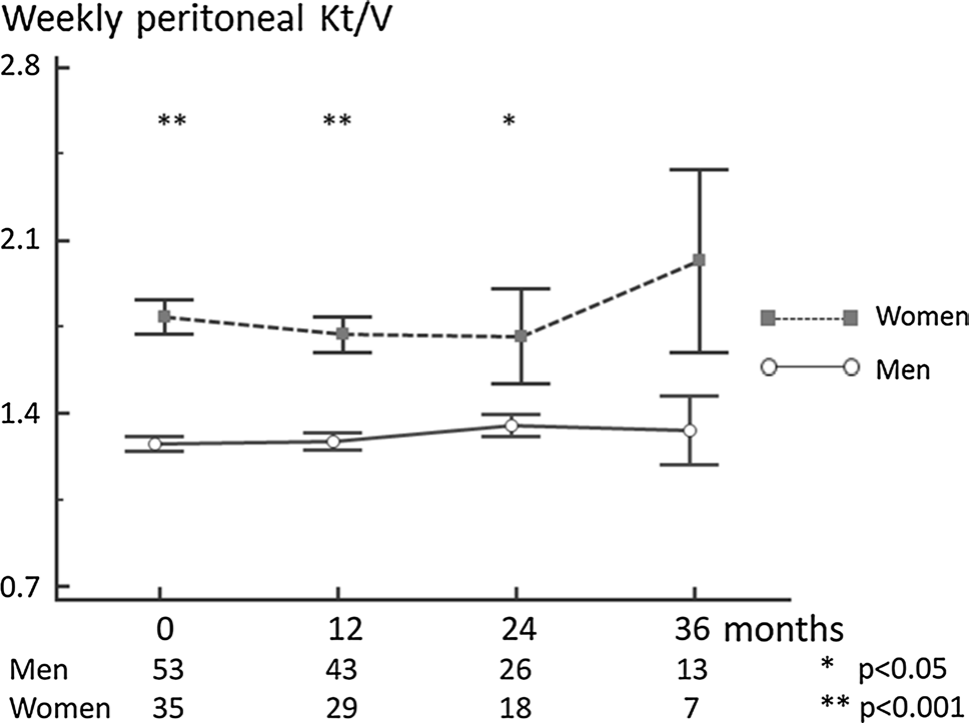
Peritoneal Kt/V in 88 incident PD patients

During the first year of the observation period, the total Kt/V was significantly higher in women; Fig. 3. This was predominantly due to the differences in peritoneal Kt/V which was much higher in women, in comparison with men. This significant difference persisted through the observation period; Fig. 4. The mean peritoneal Kt/V throughout the 36 months of observation was 28% higher in women, as compared to men.

Haemoglobin concentrations turned out to be related to the dialysis adequacy, as assessed by the total Kt/V. After 12 months of dialysis treatment, the correlation coefficient for Hb level and Kt/V equalled *r* = 0.26; *p* < 0.05. This association was similarly positive throughout the observation period, although it did not reach statistical significance at any other time point. As in prevalent patients, there was a negative relationship between the total Kt/V and prescribed ESA dose, although not significant at any time point of the follow-up.

It has to be underlined that despite this positive association between Kt/V and Hb, and much higher parameters of dialysis adequacy in women, they were nevertheless more anaemic and required much higher doses of ESA, compared to men.

## Discussion

The present study reveals that women treated with PD have a lower Hb concentration and are prescribed higher doses of ESA, as compared to men. This finding was confirmed in two independent groups of prevalent and incident PD patients. Moreover, the negative impact of female gender on Hb level holds true despite significantly higher total Kt/V in women, a parameter positively associated with Hb level.

Anaemia constitutes one of the major complications of ESRD. It impairs the patient’s physical endurance and causes cognitive dysfunction deteriorating quality of life. Moreover, it negatively affects cardiac function as it leads to left ventricular hypertrophy. If severe, anaemia increases the risk of death [7]. Given its detrimental effects, it is widely acknowledged that anaemia should be treated and, partly, corrected in dialysis patients. The Kidney Disease: Improving Global Outcomes (KDIGO) guidelines on anaemia management in CKD distinguishes different thresholds for men and women to diagnose anaemia [1]. These are less than 13.0 g/dl for men and below 12.0 g/dl for women. However, indications for ESA treatment as well as the target Hb concentrations are set identical for both genders.

In the present evaluation, lower Hb levels were observed in both prevalent and incident PD women. Haemoglobin was lower despite the fact that men were characterized by an increased baseline CRP and a higher prevalence of co-morbidities, two acknowledged risk factors for anaemia severity [8]. The findings of the present study stay in accordance with previous evaluations in pre-dialysis and haemodialysis patients [4, 9]. In the general population, the reference Hb values are different for men and women. It is not clear whether, indeed, women require less Hb or whether lower concentrations result from iron deficiency in view of menstrual blood losses and, perhaps, poorer dietary intake [2]. The role of hormonal differences between genders as a contributing factor has also been proposed [2]. Similarly, in haemodialysis patients, decreased iron due to menstruations is put forward as the main contributor by some authors [10]. However, most studies have not confirmed associations between iron status and decreased Hb in haemodialysis women [4, 11]. According to our knowledge, no studies elucidating this issue in PD patients have been reported to date. In the present evaluation, iron status was comparable between genders in the group of incident PD patients, both at baseline and during the follow-up.

ESA responsiveness and anaemia correction in dialysis patients depends on numerous factors [12–14]. One of the major ones is dialysis adequacy [4]. Uremic toxicity in general and/or retention of some direct inhibitors of erythropoiesis results in aggravated anaemia and poor response to ESA treatment. The present study confirmed this association, as the Hb concentrations turned out to be related to the total Kt/V in both studied cohorts. Moreover, in the prevalent group, Kt/V was negatively associated with ESA dose.

In the present evaluation, women were characterized by a significantly higher Kt/V, both in the prevalent and incident group. In incident patients, this was due to significantly higher dialysis dose, as reflected by peritoneal Kt/V throughout the observation period. Obviously, women, with their volume of urea distribution being lower than in men, are expected to achieve higher Kt/V when prescribed the same dialysis dose [15].

However, in the present study, women, despite reaching higher adequacy parameters, presented with lower Hb concentrations, in comparison with men. Perhaps, it is worthy of consideration to aim for a higher Kt/V in women in order to alleviate anaemia. On the other hand, it is well acknowledged that increasing dialysis dose above the currently recommended threshold does not lead to improved outcome [16].

Having lower Hb concentrations, women receive more ESA. It is well acknowledged that ESA treatment can be associated with an increase in blood pressure and with an enhanced risk of thrombotic complications [17]. Moreover, the ESA resistance index (ERI), defined as a ratio between the ESA dose and achieved Hb, is associated with a higher risk of death in haemodialysis patients [18]. Although studies in PD patients have not shown such associations [19], the question arises as to whether it is necessary to increase ESA dose in women in order to achieve Hb concentrations comparable to Hb levels in men. It is a matter of debate and, perhaps, future studies to establish whether the increased ERI in women, observed in the present evaluation, reflects hyporesponsiveness to ESA or just physiologically lower Hb concentration. The issue is not only of clinical, but also of financial importance, as increasing the ESA doses in women to achieve Hb levels comparable to men is an expensive approach.

Our study has its limitations that ought to be underlined. First, it is an observational analysis; therefore, causal relationships cannot be assessed. Secondly, no information on the menstrual status of the studied women was obtainable. However, the comparable iron status between genders in the studied group suggests that the potential impact of menstruation on observed Hb levels is negligible. The Registry does not include data potentially influencing Hb concentration and/or dialysis adequacy, as CRP or BMI. The study has also its strengths. It has been conducted on most numerous group of PD patients in Poland so far, utilizing data from the national Registry of PD. The major findings have been verified and confirmed in incident PD patients, in a longitudinal 36-month long evaluation.

 In conclusion, we have demonstrated that there is a gender-dependent difference in Hb concentration of patients treated with PD with women demonstrating significantly lower Hb despite better dialysis adequacy. This results in much higher doses of ESA prescribed. Future studies should clarify whether this approach is correct.
